# A case report of case report pursuit by medical student

**DOI:** 10.15694/mep.2019.000073.1

**Published:** 2019-04-01

**Authors:** Jonathan Li, Jennifer Wilson, Wayne Bond Lau

**Affiliations:** 1 Sidney Kimmel Medical College; 2 Office for Professional Writing; 3 Department of Emergency Medicine

**Keywords:** Undergraduate Medical Education, Case Report, Mentorship, Medical Student, Academic Medicine, Medical Teaching, Communication

## Abstract

This article was migrated. The article was marked as recommended.

Medical students often seek case reports as vehicles for academic writing opportunities, conference presentation avenues, and residency/fellowship application highlights. Here we review a case where, due to unfortunate circumstances, a student made a unique diagnosis central to proper patient clinical care, wished to write up the case subsequently, but was ultimately excluded from the final work stemming from the patient case. We review the pitfalls that occurred in the process of pursuing publication of an interesting case, the educational value of pursuing case reports for students, the necessity for strong mentorship in this process, and general principles that medical students can follow regarding case report creation to avoid being “burned”.

## Introduction

Medical students often seek case reports as vehicles for academic writing opportunities, conference presentation avenues, and residency/fellowship application highlights. Here we review a case where, due to unfortunate circumstances, a student made a unique diagnosis central to proper patient clinical care, wished to write up the case subsequently, but was ultimately excluded from the final work stemming from the patient case. Below, we will review the pitfalls that occurred in the process of pursuing publication of an interesting case and general principles that medical students can follow regarding case report creation to avoid being “burned”. The medical specifics of this case will be generalized to maintain anonymity.

## Case

A third-year medical student, on a mandatory inpatient rotation, was assigned to care for a patient with an unusual constellation of neurologic symptoms. The attending physician noted several distinct, unusual characteristics of the patient’s presentation, but given limited non-diagnostic lab results and imaging, the team was unable to reach a definitive diagnosis. After independently performing an extensive literature search, the medical student eventually found a disease reflecting the patient’s presentation and physical examination, with a notable exception of pre-existing disease for which the diagnosis was a rare complication.

The student then performed a focused review and identified 20+ published case reports concerning the diagnosis, and developed a spreadsheet to compare these reports. When approached by the student about the possible diagnosis, the attending physician did not feel strongly about the suggestion. When presented the student’s review, the team’s senior resident agreed with the potential diagnosis. After discussing it privately with the attending physician, the senior resident was able to convince the attending physician to consult a specialist team.

A fellow from the consultant team assessed the patient. The medical student discussed the potential diagnosis with the fellow, and provided the consultant team with printouts of the case reports. The fellow informed the medical student that the patient could not possibly have such a diagnosis, because it was a rare complication of a disease the patient did not have. With time, both attending physicians of the primary and consultant team decided to include the rare diagnosis in the differential. A biopsy was performed, and confirmed the rare diagnosis. Definitive treatment could now be initiated.

At this time, the medical student proposed write up of the case with the primary team attending physician. Enthusiastic at the time, the attending physician agreed it was worthy for publication since it would be the first presentation of this disease without antecedent disease history. However, the attending physician stipulated any write-up should wait until evidence the patient responded to therapy. One week later, the attending physician rotated off service. The student completed the inpatient rotation. Two months later, the team’s senior resident completed residency training. The medical student continued to follow the patient’s treatment course and kept communication with the attending physician every 2 months. Four months later, the patient was found to have responded to treatment, but the student was unable to reach the attending to confirm the next step in writing up the case.

Five months after initial accurate diagnosis of the patient, a different resident reached out to the student about writing up the case as an abstract for presentation at a national conference. The student responded, but still pursued writing the case as a manuscript, and could not receive confirmation from the resident or the initial attending physician. When the student happened to rotate through the consultant service that same month, he discussed the unique case with the specialist attending physician. To the student’s surprise, the attending informed him that the fellow originally consulted for the patient was writing up the case for publication. The student reached out to the fellow. The fellow responded that the case write-up was complete, and there was nothing the student could do to help.

The medical student reached out again to the attending physician of the primary team, who acknowledged awareness of the case report authored by the consultant team. The attending physician had been asked to review a component of it prior to publication, and thus was included in the author list. The medical student was not included in the authorship. The medical student then sought guidance from a third-party mentor not involved in the case. The medical student was advised that as a member of the inpatient service, the primary attending is the acting representative for the student with the consulting team. The student, cautiously restated his desired involvement in the manuscript and requested to be advocated for by the primary attending. The attending stated there was little to do at this point, as it appeared that the consultant service conducted its own background and literary search, and in the situation of clinical case reports co-managed by multiple specialties, “it is a race to publication”.

## Discussion

Case reports provide medical students and physicians-in-training the opportunity to begin engaging in simpler scientific medical writing before pursuing more advanced forms of medical writing (e.g. research manuscripts, book chapters). Case reports also afford an early opportunity to publish outside of formal scientific research projects. ( Har-el, 1999; Mishra, 2015) They engage a pertinent clinical question, and give students practice in research and assessment skills that forge strong clinicians. ( Florek and Dellavalle, 2016) Packer et al. identified five educational benefits of case reports for medical students ( Packer et al., 2017):

1. Developing observation and pattern recognition skills. Students who engage in writing case reports become better at recognizing and understanding nuances of disease presentations.
2. Developing hypothesis-generating skills. Students learn self-criticism and hone hypothesis-generating skills through engaging in discourse, refining arguments, and anticipating criticisms.
3. Understanding patient-centered care. Students appreciate the individual variations in disease presentation, progression, and treatment outcomes because case reports focus upon an individual patient.
4. Writing skills and rhetorical versatility. Students engage in the four classical rhetorical modes of narrative, descriptive, expository, and argumentative writing, which ultimately improves communication skills in all aspects of medicine.
5. The case report as a “mini-thesis”. Students answer a clinical question, support a possible answer, and contribute their synthesis to the scientific literature body.

Despite these advantages, medical students face major challenges and barriers in the process of writing case reports. In one survey-based study examining writing and presenting case reports among 84 fourth-year medical students, only approximately one-third had written or presented a case report. Almost all (99%) believed that finding a good mentor was a key component to finding success in the process, while major perceived barriers included a lack of formal training and the lack of a mentor. ( Jha et al., 2018) This study highlights the essential role solid mentorship plays in supporting engaged, motivated students choosing to write case reports. The study also corroborates clearly the significant challenges encountered by the student in the case above.

Mentoring and role modeling are critical components of the formal, informal, and hidden medical education curriculum. Poor role modeling experiences can leave lasting negative impacts on students and change student behavior to become aversive towards similar situations ( Mileder, Schmidt and Dimai, 2014). Thus, when students experience poor role modeling related to a fundamental form of medical literature, it may have profound effects upon their future, including career decisions. ( Mileder, Schmidt and Dimai, 2014)

In the case above, did mentors exhibit poor role modeling in failing to include the student in co-authoring the report. Authorship issues are a common source of conflict during the pre-publication stage. The position of the first author is highly coveted and reflects an individual’s time and dedication towards manuscript preparation and publication. The last author of the paper identifies the senior individual providing guidance and oversight. Universities have additional guidelines for determining who else is a co-author. Our institution recommends steps to avoid authorship disputes, with a focus on clearly defining the role of each author and on early, frequent, and open communication during the drafting process.(Office of Research Conduct and Compliance, Thomas Jefferson University, 2017)

If a dispute occurs, the research group should first attempt to resolve the conflict internally. If the disagreement remains unresolved, then they should consult a senior third-party colleague. If all else fails, refer to institutional policy and involve senior administrative level colleagues. The International Committee of Medical Journal Editors (ICMJE) provides guidelines to determine how individuals qualify for authorship and to prevent gift authorships and unethical distribution of credit. Authors should meet all of the following three conditions: 1) Make substantial contributions to conception and design, acquisition of data, or analysis and interpretation of data; 2) draft the article or revise it critically for important intellectual content; and 3) provide final approval of the version to be published. Those who are involved with the manuscript but do not meet these criteria should be listed in the acknowledgments section. ( Gopikrishna, 2010; Rison, 2013)

The medical student in the presented case would not have met authorship criteria based on clinical involvement alone, but might have been listed in the acknowledgments section. Had the primary attending physician advocated for the student to be included in the writing process, however, the student would have had an opportunity to meet criteria for authorship. For instance, given the continued interest exhibited by the student through consistent communication, the primary attending physician could have established early communication between the specialty service already writing the patient case and the motivated medical student. And when the specialty service initially contacted the primary attending to review the component of the patient case report, the primary attending could have advocated at that junction on behalf of the medical student.

As exemplified in this case, medical students cannot necessarily rely on the advocacy of attending physicians. Any student interested in writing up a potentially interesting clinical case is advised to immediately approach all involved parties and constantly engage in communication from the onset of idea conception. It is not sufficient to only have dialogue with the primary team members, as evidenced in this case. One party’s reluctance to engage in an academic written activity should serve as impetus for student communications with other involved team members, to secure concrete mentoring in project development. Dialogue should be frank, and conversations should be documented to confirm responsibilities of all parties involved. Early, inclusive communication is critical to increase the number of potential mentors for manuscript production, establish early leadership in a project’s development, and prevent unpleasant late surprises.

## Conclusion

Teaching faculty should support medical students who are interested in writing case reports because the process provides students with unique educational opportunities. Lack of quality mentorship is a major barrier for medical students pursuing case report completion. To increase mentor/mentee relationships resulting in concrete academic accomplishments, students should contact multiple teaching faculty involved in the case early and engage in communication amongst all potential co-authors. Identification of a communicative mentor, discussing authorship, dividing project responsibilities early, and maintaining frequent communication through all stages of academic production is central to a student’s success in writing a case report.

## Take Home Messages

• Medical students should express interest early in pursuing a case report and identify supportive and communicative mentors.
• Communication should be established early between all parties involved in the care of the patient and maintained frequently throughout production of the manuscript.
• Authorship and project responsibilities should be discussed early.
• Case reports are unique educational opportunities for medical students and mentorship is an essential component of this learning experience.
• Mentors should maintain open communication with students.

## Notes On Contributors

Jonathan C. Li, BS is a fourth-year medical student at Sidney Kimmel Medical College at Thomas Jefferson University. He is pursuing a career in Combined Internal Medicine and Pediatrics.

Jennifer Fisher Wilson, MS, ELS, is the Senior Writer/Editor for the Office for Professional Writing, Publishing, and Communication at Jefferson (Philadelphia University+Thomas Jefferson University). She is a former writer for Annals of Internal Medicine and The Scientist.

Wayne Bond Lau, MD is Professor of Emergency Medicine at Thomas Jefferson University Hospital. He contributes to EM through clinical care, basic research (cardiac ischemia/reperfusion injury), and education of medical students/residents at Jefferson. As Director of the Jefferson Chinatown Clinic, he has cared for Philadelphian underserved communities for over a decade.

## References

[ref1] Florek A. G. and Dellavalle R. P. (2016) Case reports in medical education: a platform for training medical students, residents, and fellows in scientific writing and critical thinking. Journal of Medical Case Reports. 10, p.86. 10.1186/s13256-016-0851-5 27048362 PMC4822269

[ref2] Gopikrishna V. (2010) A report on case reports. Journal of Conservative Dentistry: JCD. 13(4), pp.265–271. 10.4103/0972-0707.73375 21217956 PMC3010033

[ref3] Har-el G. (1999) Does it take a village to write a case report? Otolaryngology--Head and Neck Surgery: Official Journal of American Academy of Otolaryngology-Head and Neck Surgery. 120(6), pp.787–788. 10.1016/S0194-5998(99)70314-1 10352427

[ref4] Jha P. Thakur A. Klumb J. and Bhandari S. (2018) Perceptions of Fourth-Year Medical Students on Writing and Presenting Case Reports. Cureus. 10(3), p. e2341. 10.7759/cureus.2341 29805923 PMC5963942

[ref5] Mileder L. P. Schmidt A. and Dimai H. P. (2014) Clinicians should be aware of their responsibilities as role models: a case report on the impact of poor role modeling. Medical Education Online. 19, p.23479. 10.3402/meo.v19.23479 24499869 PMC3916672

[ref6] Mishra S. (2015) Are medical breakthroughs declining - The importance of case reports? Indian Heart Journal. 67 Suppl 3, pp.S1–S3. 10.1016/j.ihj.2016.01.005 PMC4799008 26995410

[ref7] Office of Research Conduct and Compliance, Thomas Jefferson University (2017) GUIDELINES FOR AVOIDING AND RESOLVING AUTHORSHIP DISPUTES. Available at: https://www.jefferson.edu/content/dam/university/research/compliance/Guidelines%20for%20Avoiding%20Resolving%20Authorship%20Disputes.pdf( Accessed: 2 February 2019).

[ref8] Packer C. D. Katz R. B. Iacopetti C. L. Krimmel J. D. (2017) A Case Suspended in Time: The Educational Value of Case Reports. Academic Medicine: Journal of the Association of American Medical Colleges. 92(2), pp.152–156. 10.1097/ACM.0000000000001199 27097050

[ref9] Rison R. A. (2013) A guide to writing case reports for the Journal of Medical Case Reports and BioMed Central Research Notes. Journal of Medical Case Reports. 7, p.239. 10.1186/1752-1947-7-239 24283456 PMC3879062

