# Supplementary material for: A case report of case report pursuit by medical student
Source: MedEdPublish (2016). 2019 Apr 1;8:73. [Version 1] doi: 10.15694/mep.2019.000073.1 (PMC10712637; doi:10.15694/mep.2019.000073.1)
Supplement: Peer review history

# Reviewer report: Reviewer response for version 1

**DOI:** 10.21956/mep.19823.r29860

**Published:** 2019-06-10

**Authors:** Trevor Gibbs

**Affiliations:** 1 AMEE

This review has been migrated. The reviewer awarded 4 stars out of 5 A well written and interesting paper that describes some of the pitfalls that students can fall into in their pursuit of scholarship, and some of the difficulties arising from lack of recognition by faculty. It points to some salient issues relating to the faculty- student relationship. This well written and descriptive paper prvides some possible answers that we all need to take notice of.I fully recommend this paper to all those involved with mentoring or supervision of students

Reviewer Expertise:

NA

No decision status is available

# Reviewer report: Reviewer response for version 1

**DOI:** 10.21956/mep.19823.r29861

**Published:** 2019-04-05

**Authors:** Gerard Flaherty

**Affiliations:** 1 National University of Ireland Galway

This review has been migrated. The reviewer awarded 4 stars out of 5 This is a novel and important manuscript and one which deserves as wide an audience as possible in my opinion. The authors are to be commended for addressing an important issue in medical education with balance and professionalism. Their case study highlights the often vulnerable position of medical students in the training hierarchy. According to principles of natural justice, the medical student in the case described should have been afforded the opportunity to contribute to or even to prepare the first draft of the case report, based on a diagnosis first suggested by that same student. Unfortunate timing with lack of continuity of clinical staff, questionable role modelling and the complete lack of mentorship conspired to prevent the student from realising a worthy ambition of co-authoring a case report with which he/she was intimately involved. I feel only admiration for the student involved and hope that this encounter with the sometimes dysfunctional power relationships in clinical practice will not discourage future publication attempts. This case study is very well written and well supported by reference to the literature. The set of recommendations provided will itself be an important contribution to the medical education literature and I believe that it will help in the creation of ‘ground rules’ which will serve to guide clinical teams who may face similar situations in future. We must cherish our learners and support them in their efforts to advance and grow in knowledge. The opposite, unfortunately, occurred in the case described but lessons have luckily been learned.

Reviewer Expertise:

NA

No decision status is available
